# Influence of a Mixture of Protein Hydrolysate from Black Soldier Fly Larvae and Schizochytrium on Palatability, Plasma Biochemistry, and Antioxidative and Anti-Inflammatory Capacity in Cat Diets

**DOI:** 10.3390/ani14050751

**Published:** 2024-02-28

**Authors:** You Li, Mingkang Liu, Yu Wei, Luyang Li, Deying Ma, Yuxiao Weng, Haifeng Wang, Xiao Xu

**Affiliations:** 1 Hubei Key Laboratory of Animal Nutrition and Feed Science, Wuhan Polytechnic University, Wuhan 430023, China; 19107133348@163.com (Y.L.); 15527199297@163.com (M.L.); yuwei20000306@163.com (Y.W.); 17371453782@163.com (L.L.); 15181488346@163.com (D.M.); 2 P&O Biotechnology (Hubei) Co., Ltd., Wuhan 436043, China; 13707162068@139.com (Y.W.); 18627780422@126.com (H.W.)

**Keywords:** protein hydrolysate, black soldier fly larvae, schizochytrium, palatability, anti-inflammation, cats

## Abstract

**Simple Summary:**

Protein hydrolysate has been a premium protein source in animal feed and is more easily absorbed in animal intestines compared with crude protein, which has a higher rate of pure protein and free amino acids with a high protein efficiency ratio. Previous studies have shown that black soldier fly larvae have been considered one of the most promising proteins for replacing fish meal, especially in the pet market. Recently, the synthesis and functional identification of protein hydrolysate from black soldier fly larvae (BSFP) have received a great deal of attention. In addition, schizochytrium is a marine microalga which has a high content of lipids and polyunsaturated fatty acids (PUFAs), especially an abundant docosahexaenoic (DHA) concentration. Previous research has demonstrated that schizochytrium is promisingly able to substitute for soybean and fish oil in feed and possesses some physiological functions.

**Abstract:**

The objective of this research was to evaluate palatability, plasma biochemistry, antioxidative and anti-inflammatory capacity, and immune levels in cats by feeding supplementing inclusion of different levels of a mixture of protein hydrolysate from black soldier fly larvae and schizochytrium (BSFPs) in diets. In the feed experiment, a total of 24 adult cats (12 females and 12 males; BW: 3.02 ± 0.06 kg) were randomly divided into four groups: (1) diet with chicken and fish meal as primary protein resource (CON); (2) diet with 5% BSFPs replacing chicken meal, fish meal, chicken oil, and fish oil (5% BSFPs); (3) 10% BSFPs; and (4) 15% BSFPs. The body weight and feed intake were recorded, and a blood sample was collected for analysis. In the palatability experiment, three diets containing 5%, 10%, and 15% BSFPs were evaluated by comparing with CON. These results suggested that different levels of BSFPs could improve palatability in cat diets by enhancing the first sniff, the first bite, and feed intake (*p* < 0.05). However, no significant influence existed in body weight and average daily feed intake (*p* > 0.05). In comparison to the CON group, 5% and 15% BSFPs significantly increased the total protein content, and all treatment groups decreased the triglyceride content and enhanced the calcium concentration in plasma; in addition, the activity of aspartate aminotransferase and alanine aminotransferase and the content of creatinine and urea nitrogen were significantly reduced by the supplementation inclusion of BSFPs in the diets (*p* < 0.05). The enzyme activity of glutathione peroxidase was dramatically enhanced by the supplementation of 10% and 15% BSFPs in diets compared with the CON diet, and the activity of superoxide dismutase was increased and the malondialdehyde concentration was remarkably reduced in all three treatments (*p* < 0.05). Compared with the CON group, different levels of BSFPs in the diets significantly increased the immunoglobulin A content in plasma; similarly, the immunoglobulin G concentration was significantly enhanced by the supplementation of 10% and 15% BSFPs in the diets (*p* < 0.05). Furthermore, the interleukin-1β content was significantly reduced in the inclusion of 10% and 15% BSFPs in the diets, and 15% BSFPs remarkably decreased the content of interleukin-8 in plasma compared with the CON diet (*p* < 0.05). To sum up, the supplementation of different levels of BSFPs exhibited a positive effect on palatability and enhanced the antioxidant, anti-inflammatory, and immune capacity. Particularly, the addition levels of 10% and 15% BSFPs were more effective in antioxidation, anti-inflammation, and immunity.

## 1. Introduction

Pet companions are getting increasing attention and affection, and the pet food market is also constantly growing. Due to its huge size, sustainable development is a serious problem, especially in ingredient selection and nutritional content [1]. Among nutrients, protein is the most expensive and ecologically demanding macronutrient [2]. With the increase in human and animal populations and the vigorous development in the number of pets, protein resources such as fish meal and soybean meal, which can have serious shortages, cannot meet the growing needs of the pet industry [1,3]. This is driving the development of new high-quality and sustainable protein sources for pet food, especially for carnivorous cats.

Proteolysis is the process of the partial or substantial hydrolysis of proteins to produce oligopeptides, polypeptides, and free amino acids [4]. Compared with crude protein, the protein hydrolysate can be more easily absorbed in animal intestines because of a higher rate of peptides and free amino acids and also a high protein efficiency ratio [5,6]. In addition, it has a strong ability to inhibit the peroxidation of micro-biomolecules and free radicals with the many antioxidative peptides in the protein hydrolysate [7], and some bioactive peptides, produced by previous in vitro protein hydrolysis, also have beneficial biological activity, such as anti-inflammatory, antihypertensive, and hypocholesterolemic activity [8]. Black soldier fly larvae have been considered one of the most promising proteins for replacing fish meal due to their high protein content, fertile amino acid composition, and rich minerals and vitamins [9]. In recent years, the synthesis and functional identification of the protein hydrolysate from black soldier fly larvae (BSFP) has received a great deal of attention. For example, the protein hydrolysate from black soldier fly larvae was hydrolyzed by using a bromelain enzyme to produce a high amount of essential amino acids, such as lysine, leucine, and valine, and enhance the activity of antioxidation [10]. Moreover, a study showed that the protein hydrolysate has substantial antioxidant activity, which can reduce reactive oxygen species (ROS) production and relieve oxidative stress in L-929 cells caused by H_2_O_2_ [11].

Polyunsaturated fatty acids (PUFAs) are important nutrients for animal growth and survival by providing an energy source. The inclusion of omega 3 (n-3) PUFAs in diets can improve the nutritional quality, modulate the inflammatory response, and reduce the risk of cancer [12]. In particular, docosahexaenoic (DHA, 22:6, n-3) fatty acids are more important, with some function of enhancing neurodevelopment, cognitive ability, and anti-tumor capacity [13,14]. Schizochytrium is a marine microalga which has a high content of lipids and PUFAs, especially an abundant DHA concentration [15]. In addition, schizochytrium is a promising potential alternative to conventional n-3 PUFA sources, with a high potential for production and lower processing costs compared with fish oil [16]. Early research showed that supplementation of schizochytrium at 0.75% can improve the growth performance, immune response, and intestinal health of penaeus monodon (a species of shrimp) [17]. In addition, schizochytrium could enhance the oxidative stability of dairy products with natural antioxidative compounds [18]. Furthermore, the addition of 0.4% schizochytrium in the diet could enhance palatability and increase the digestibility of nutrients and the phagocytic cell numbers of dogs [19].

Therefore, the objective of this study was to evaluate the diet palatability, plasma biochemistry, immune level, and antioxidative and anti-inflammatory capacity in cats by feeding diets including different levels of supplementing a mixture of protein hydrolysate from black soldier fly larvae and schizochytrium (BSFPs) to partially replace chicken meal, fish meal, chicken oil, and fish oil.

## 2. Materials and Methods

### 2.1. Experimental Animals and Design

The animal trial was conducted according to the Animal Scientific Procedures Act 1986 (Home Office Code of Practice. HMSO: London, UK, January 1997) and EU regulations (Directive 2010/63/EU). The experimental protocol (No. WPU202306059) used in this study was approved by the Institutional Animal Care and Use Committee of Wuhan Polytechnic University (Wuhan, China).

#### 2.1.1. Feed Experiment

A total of 24 adult cats of similar health and weight (12 females and 12 males; BW: 3.02 ± 0.06 kg) were randomly divided into 4 treatments consisting of 6 replicates per treatment and one cat per replicate, which was housed in a single cage. Four treatment diets included the following: (1) a basal diet group (CON group); (2) a basal diet supplementing with 5% of a mixture of protein hydrolysate from black soldier fly larvae and schizochytrium to replace chicken meal, fish meal, chicken oil, and fish oil (5% BSFPs group); (3) a basal diet supplementing with 10% of a mixture of protein hydrolysate from black soldier fly larvae and schizochytrium to replace chicken meal, fish meal, chicken oil, and fish oil (10% BSFPs group); and (4) a basal diet supplementing with 15% of a mixture of protein hydrolysate from black soldier fly larvae and schizochytrium to replace chicken meal, fish meal, chicken oil, and fish oil (15% BSFPs group). On the basis of the Association of American Feed Control Officials [20], the diets were formulated to satisfy the nutrient requirements of adult cats in Table 1. The trial lasted for 33 days, with the first 5 days as the adaptation period and 28 days as the trial period. The mixture of protein hydrolysate from black soldier fly larvae and schizochytrium (BSFPs) was a commercial product provided by P&O Biotechnology (Hubei) Co., Ltd., Ezhou, China. The ratio of BSFP hydrolysate to schizochytrium was 4:1, and the nutritional composition is shown in Table 2.

#### 2.1.2. Palatability Experiment

According to the international palatability comparison test method “Two-Bowl Test”, three experimental groups (5% BSFPs, 10% BSFPs, and 15% BSFPs) were assessed for palatability versus the basal diet (CON) in cats [21]. Every experiment was implemented using a split-plate test, where cats were given 40 g of diet in each of two stainless steel bowls for 30 min. Each comparative experiment including a total of 18 cats was conducted in 2 continuous periods with 4 days per period, and the position of the bowl was changed every day. The detailed operation abided by the previous method with a slight change [22]. Each cat’s “first sniff” and “first bite” were the first food bowls they touched and ate, respectively, and the relative ratio was calculated. Based on the amount of each diet each cat ate in a 30 min period, the feed intake of the diet was calculated.

First sniff (%) = number of Diet A/(total number of Diet A + Diet B)(1)

First bite (%) = number of Diet A/(total number of Diet A + Diet B)(2)

### 2.2. Sample Collection

Every cat was individually weighed at 08:00 h on the first day and the 28th day, and the initial body weight (IBW) and the final body weight (FBW) were respectively recorded. During the experiment period, the average daily feed intake (ADFI) was calculated by collecting the feed intake. After weighing the FBW, blood samples were collected from the jugular vein of each cat with centrifuging at 3500 rmp for 10 min, and plasma was extracted and stored at −80 °C for analysis.

### 2.3. Plasma Biochemical Parameters

Some plasma biochemical indicators, including total protein (TP), albumin (ALB), glucose (GLU), triglyceride (TG), total cholesterol (TC), calcium (CA), phosphorus (P), aspartate aminotransferase (AST), alanine aminotransferase (ALT), creatinine (CREA), and urea nitrogen (BUN), were measured with an automatic biochemical analyzer (7100, HITACHI, Tokyo, Japan). According to previous research, the plasma biochemical parameters were considerately assessed, and the concrete reference interval was exhibited in the result [23].

### 2.4. Antioxidative Capacity

The total antioxidant capacity (T-AOC, A015-2-1), glutathione peroxidase (GSH-PX, A005-1-2), superoxide dismutase (SOD, A001-1-2) enzyme activity, and malondialdehyde (MDA, A003-1-2) content in the plasma were evaluated with a kit produced by the Nanjing Jiancheng Institute of Biological Engineering (Nanjing, China).

### 2.5. Inflammatory Cytokines and Immune Levels

According to the manufacturer’s instructions, pro-inflammatory cytokines, including tumor necrosis factor-α (TNF-α, RX1600891C), interleukin-8 (IL-8, RX1600893C), and interleukin-1β (IL-1β, RX1600890C), and immunoglobulin levels, such as immunoglobulin A (IgA, RX1600887C), IgG (RX1600884C), and IgM (RX1600885C), were measured using feline enzyme-linked immunosorbent assay (ELISA) kits provided by Quanzhou Ruixin Biotechnology Co., Ltd., Quanzhou, China.

### 2.6. Statistical Analyses

In the feed experiment, all data from the total of 24 cats were analyzed using a one-way ANOVA of SAS 9.1 software (SAS Inst. Inc., Cary, NC, USA) and shown as means and standard error of the mean (SEM). The significant level of datum was declared at *p* < 0.05. If significant effects were found, individual means were compared using Duncan’s multiple comparison tests.

All data were preliminarily processed by Excel, and the first sniff, the first bite, and the intake ratio were analyzed by a *t*-test. The significant level of datum was declared at *p* < 0.05, and the extremely significant level of datum was declared at *p* < 0.001. A total of 18 cats were considered the experiment units for analysis.

## 3. Results

### 3.1. Body Weight

Compared with the CON group, the diets that supplemented different levels of protein hydrolysate from black soldier fly larvae and schizochytrium had no significant difference on the BW at 28 days for cats in Table 3 (*p* > 0.05). Meanwhile, a similar consequence was shown in the ADFI that there was no significant difference (*p* > 0.05).

### 3.2. Plasma Biochemistry Indicators

As shown in Table 4, there was a significant influence that the inclusion levels of 5% BSFPs and 15% BSFPs in cat diets can significantly enhance the TP content in plasma compared to the CON group (*p* < 0.05). Compared to the CON group, supplementation of different levels of BSFPs in diets significantly decreased the TG content and increased the CA concentration in the plasma for cats (*p* < 0.05). Furthermore, the content in the plasma of AST, ALT, CERA, and BUN were significantly reduced by supplementing inclusion levels of BSFPs in diets compared with feeding the CON diet (*p* < 0.05).

### 3.3. Antioxidative Capacity

Table 5 demonstrates that the plasma T-AOC had no significant difference among the cats fed the diets supplemented with inclusion levels of BSFPs compared to the CON diet (*p* > 0.05). However, compared with the CON group, the addition of 10% BSFPs and 15% BSFPs in diets significantly enhanced the activity of GSH-PX in the plasma (*p* < 0.05). In addition, the activity of SOD was significantly increased and the concentration of MDA was significantly decreased in the plasma by supplementing inclusion levels of BSFPs in cat diets compared to the CON group (*p* < 0.05).

### 3.4. Infammatory Cytokines and Immune Levels

The content of TNF-α in the plasma had no significant difference between all treatments and the CON group in Table 6 (*p* > 0.05). Nevertheless, compared with the CON group, the 10% BSFPs and 15% BSFPs groups significantly reduced the concentration of IL-1β, and the content of IL-8 was significantly decreased by supplementing 15% BSFPs in the diets (*p* < 0.05). Moreover, the supplementation of different levels of BSFPs in the diets significantly enhanced the content of IgA in the plasma, and the concentration of IgG was significantly increased by feeding inclusion levels of 10% BSFPs and 15% BSFPs diets compared to the CON diet in Table 7 (*p* < 0.05).

### 3.5. Palatability

Table 8 shows the results of the palatability test between different levels of BSFPs diets and a commercial cat diet. Through two continuous days of palatability test, the first sniff, the first bite, and the feed intake were significantly promoted by feeding inclusion of different levels of BSFPs in diets compared to the CON diet (*p* < 0.05).

## 4. Discussion

Generally speaking, soybean, chicken, swine, fish meat, and bone byproducts are the main protein sources for the pet market, and corn, sunflower, soybean, fish, and chicken oils are usually used as oil sources for pet animals [1,24]. However, the traditional resources of protein and oil have failed to meet the increasing demands of the pet industry. Recently, black soldier fly larvae have been reported as a novel protein source for a substitute to replace fish meal [25], and the protein hydrolysate from enzymatically hydrolyzed black soldier fly larvae has improved the antioxidative capacity, which can relieve oxidative stress [10,11]. Furthermore, schizochytrium is a rich source of docosahexaenoic acid (DHA), which has been considered as an alternative to fish oil with some functions such as antioxidative, anti-inflammatory, and anti-tumor capacity [26,27,28]. Therefore, this study was conducted to explore diets supplementing different levels of a mixture of protein hydrolysate from black soldier fly larvae and schizochytrium (BSFPs) on palatability, plasma biochemistry indicators, and antioxidative and anti-inflammatory capacity in cats.

According to survey results, the cat obesity ratio was constantly increasing, reaching above 30–40%, which has been relevant to health problems, such as renal disease, skin disease, and diabetes mellitus [29]. Body weight and feed intake are crucial indexes of growth performance associated with the healthy body of cats [30]. In this research, the cats fed with inclusion levels of BSFPs had no negative influence on the FBW and the ADFI. Consistent with previous studies, protein hydrolysate that originated from insect enzymatic hydrolysis, such as mealworm and superworm, had no significant difference on growth performance in sea trout [30], and replacing chicken meal with 20% BSFP showed no negative effect on growth performance in beagle dogs [31]. Furthermore, the supplementation of schizochytrium in diets had no significant difference on average gain weight and gain to feed for growing lambs [32], and the substitution of fish oil with schizochytrium in diets for juvenile rainbow trout had no effect on growth performance [33]. Therefore, supplementation with a mixture of protein hydrolysate from black soldier fly larvae and schizochytrium (BSFPs) in diets had no negative influence on body weight and induced obesity for cats. In the current study, it is regretful that body condition parameters such as the body fat level were not measured. In future studies, these parameters should be determined.

The blood biochemistry parameter is a key indicator of health, and many factors such as nutrition, disease, and the environment can lead to changes [34]. This study was conducted to research the effect of supplementation inclusion levels of BSFPs on health in cat diets by analyzing the plasma biochemical indicators. Our results suggest that 5% and 15% BSFPs enhanced the TP content and that the TG content was reduced and the CA content was increased by the supplementation of different levels of BSFPs in the diets. Some studies showed similar results; supplementation of 2.5% and 25% black soldier fly larvae can respectively improve TP content in Brahma chickens and Clarias gariepinus [35,36]. The protein hydrolysate from black soldier fly larvae can be more easily absorbed in animal intestines compared with the crude protein, which has a higher rate of pure protein and free amino acids and also a high protein efficiency ratio [5,6]. The protein hydrolysate from enzyme-hydrolyzed mealworm exhibited the function of decreasing the TG content [37], and the diet added schizochytrium, which also reduced the TG content and showed the potential ability to increase the CA content in plasma [38,39]. Schizochytrium can decrease levels of plasma TG that may be associated with polysaccharides, which was abundantly produced in marine schizochytrium [40]. The content of enzyme activities, such as AST and ALT, and the content of CREA and BUN are related to the health of the liver and kidneys. Early research demonstrated that DHA effectively reduced the high-fat-diet-induced ALT and AST content and alleviated hepatic disease [41], and DHA can reduce BPA-induced nephrotoxicity by decreasing BUN and CERA concentrations in plasma [42]. However, the initial blood biochemistry parameters of the cats were not measured. It would be better to collect and determine the initial blood samples to ensure the similar health status of the cats.

Oxidative stress is caused by an imbalance of oxidative and antioxidative systems, which could accumulate an excessive amount of ROS, leading to injury to tissue and damage to DNA [43]. As a part of the antioxidant defense system, T-AOC, GSH-PX, and SOD play crucial roles in protecting cells from oxidative damage by ROS [44], and MDA is an important indicator of the oxidative state produced by ROS attacking PUFAs in membrane phospholipids [45]. In our experiment, the supplementation of 10% and 15% BSFPs in diets can enhance the activity of GSH-PX, and the activity of SOD was significantly increased and the MDA content was significantly decreased in plasma by feed inclusion of different levels of BSFPs in cat diets. Similar to our results, diets that added schizochytrium showed an antioxidative function, such as enhancing the activity of GSH-PX in beef, increasing SOD in juvenile mirror carp, and reducing the MDA content in largemouth bass [46,47,48]. Moreover, a study illustrated that schizochytrium protein hydrolysate could be a potential antioxidant additive for enhancing antioxidative capacity to treat alcohol-liver diseases [49]. In addition, some research also demonstrated that protein hydrolysate from black soldier fly larvae exhibited potential antioxidant ability [10,11].

Plasma immunoglobulins, such as IgA, IgG, and IgM, are generally used to measure cellular responses and the capacity to recognize pathogenic invasion, and a higher content can enhance the body’s defense [50]. Our research showed that the IgA concentration of plasma was significantly increased by feeding the inclusion of different levels of BSFPs in cat diets. In addition, a similar effect existed in the IgG content in which the dietary supplementation of 10% and 15% BSFPs promoted the IgG content in plasma. Previous studies showed that microalgae in animal feed will stimulate the immune system; for instance, the dietary supplementation of spirulina increased immunoglobulin concentration and immune responses [51,52]. A study illuminated that the IgG content was increased with the increasing dose of supplementation of schizochytrium in diets [53], and interestingly, the literature suggested the plasma IgA concentration had been positively influenced by marine oil [54]. Furthermore, a diet of replacing soybean meal with 25% black soldier fly larvae significantly enhanced the concentration of IgA, IgG, and IgM in the ileal of weaned piglets, and 12% black soldier fly larvae or the mixture of 12% black soldier fly larvae and 0.1% multi-probiotics can also increase the IgA and IgG content in the blood of weaned piglets [55], which was perhaps due to the lauric acid of black soldier fly larvae improving the production of immunoglobulins by relieving interleukin production [56]. Besides, chitooligosaccharides, produced by the chitin in BSFP, were able to promote immune responses [57].

Many inflammatory cytokines are relevant to inflammatory injuries such as tissue degeneration and necrosis, and the amount of production of pro-inflammatory cytokines such as TNF-α, IL-1β, and IL-8 will result in abnormal immune responses [58]. The dietary supplementation of 10% or 15% BSFPs can significantly reduce the content of IL-1β in the plasma of cats, and the IL-8 concentration was decreased by feeding the inclusion of 15% BSFPs in the diet. A work exhibited that the algal oil extracted from schizochytrium can also remarkably reduce the intestinal inflammatory response in mice treated with ceftriaxone sodium by decreasing the content of pro-inflammatory cytokines (TNF-α, IL-6, and IL-1β) [59]. In addition, adding 15% lyophilized microalgae powder from schizochytrium in the diet can maintain a normal physiological state of the intestine by downregulating the gene expression level of pro-inflammatory cytokines (IL-6, IL-8, and IL-1β) in zebrafish [60]. A similar effect exists in black soldier fly larvae as black soldier fly larvae played an anti-inflammatory function by downregulating the IL-8 and IL-1β gene expressions and mediating the mechanism of toll-like receptors (TLR) signaling [61], and the antimicrobial peptides (AMPs) from black soldier fly larvae reduced the LPS-induced nitric oxide and cytokine production in murine macrophage cells [62]. Additionally, the protein hydrolysate from black soldier fly larvae also may have anti-inflammatory activity, as other enzymatically hydrolyzed proteins of edible insects [8].

Palatability is a crucial criterion in diets and is generally used to evaluate the product performance and preference for cats, including first preference and feed intake [63]. The smell, moisture content, protein content and source, and processing ways are the main factors to determine the choice of cats [64]. In our palatability test, cats showed more affection and preference for the diets with supplementing inclusion of different levels of BSFPs by enhancing the first sniff, the first bite, and the feed intake compared with the CON diet. As a novel insect protein resource, research reported that the dietary supplementation of BSFP significantly promoted palatability for cats [65], and many works showed that protein hydrolysates are the most popular palatability enhancers in cat diets because of their high short peptide and free amino acid content [66]. Furthermore, the diet supplementing 0.4% schizochytrium positively improved the first choice and intake ratio for dogs, which showed evidence that palatability can be enhanced with high total concentrations of fatty acids [19]. In future studies, we think it is better to prolong the time of palatability measures.

## 5. Conclusions

Overall, supplementation inclusion of different levels of a mixture of protein hydrolysate from black soldier fly larvae and schizochytrium (BSFPs) in diets can positively influence palatability, enhance the antioxidative ability and immune response, and reduce the production of pro-inflammatory cytokines in cats. Above all, the levels of 10% and 15% BSFPs showed more efficient functions in antioxidation, immunity, and anti-inflammation.

## Figures and Tables

**Table 1:** Ingredients composition of cat diets containing graded inclusion levels of a mixture of protein hydrolysate from black soldier fly larvae and schizochytrium (BSFPs).

Ingredients, %	CON	5% BSFPs	10% BSFPs	15% BSFPs
Protein hydrolysate from black soldier fly larvae and schizochytrium	0.00	5.00	10.00	15.00
Chicken meal	45.00	42.00	39.00	36.00
Fish meal	15.00	14.00	13.00	12.00
Chicken liver meal	2.00	2.00	2.00	2.00
Chicken oil	8.00	7.50	7.00	6.50
Fish oil	2.50	2.00	1.50	1.00
Sweet potato granule	10.00	10.00	10.00	10.00
Tapioca flour	10.00	10.00	10.00	10.00
Apple	2.00	2.00	2.00	2.00
Distillers yeast	1.00	1.00	1.00	1.00
Flaxseed	1.00	1.00	1.00	1.00
Kelp powder	1.00	1.00	1.00	1.00
Beet pulp	1.00	1.00	1.00	1.00
Premix ^1^	1.50	1.50	1.50	1.50
Total	100.00	100.00	100.00	100.00
Nutrient level ^2^				
ME, kcal/kg	4030	4050	4020	4020
CP, %	30.5	31.5	32.0	31.5
EE, %	14.2	14.3	14.0	14.2
Ca, %	0.60	0.62	0.64	0.65
P, %	0.50	0.51	0.51	0.52

CON, basal diet group; 5% BSFPs, group with a basal diet that supplemented with 5% of protein hydrolysate from black soldier fly larvae and schizochytrium; 10% BSFPs, group with a basal diet that supplemented with 10% of protein hydrolysate from black soldier fly larvae and schizochytrium; 15% BSFPs, group with a basal diet that supplemented with 15% of protein hydrolysate from black soldier fly larvae and schizochytrium; ME, metabolic energy; CP, crude protein; EE, ether extract; Ca, calcium; P, phosphorus. ^1^ Provided per kg diet: Cu (CuSO_4_), 8.0 mg; K (KIO_3_), 0.8 mg; Fe (C_6_H_5_FeO_7_), 80 mg; Mn (MnCO_3_), 7.5 mg; Na (Na_2_SeO_3_), 154.0 mg; Zn (ZnSO_4_), 75 mg; Co (CoSO_4_), 1.5 mg; vitamin A, 6000 IU; vitamin D3, 900 IU; vitamin E, 45 IU; vitamin K3, 0.7 mg; vitamin B12, 0.03 mg; biotin, 0.07 mg; folic acid, 0.4 mg; nicotinic acid, 45.0 mg; vitamin B5, 16.80 mg; pyridoxine, 10.2 mg; riboflavin, 10.2 mg; thiamin, 10.2 mg. ^2^ The nutrient levels were calculated values.

**Table 2:** The analyzed nutritional content of protein hydrolysate from black soldier fly larvae and schizochytrium (BSFPs).

Items	Content, %	Items	Content, %
Dry matter	97.26	Arginine	2.16
Crude protein	44.84	Histidine	2.83
Crude fat	14.52	Isoleucine	1.76
Crude fiber	7.24	Leucine	2.89
Ash	19.06	Lysine	2.69
Calcium	5.70	Methionine	0.89
DHA	1.40	Phenylalanine	1.88
		Threonine	1.50
		Tryptophan	0.54
		Valine	2.46
		Alanine	3.38
		Aspartic acid	4.10
		Cystine	0.08
		Glutamic acid	5.60
		Glycine	2.33
		Proline	2.02
		Serine	1.44
		Tyrosine	2.93

**Table 3:** Effects of protein hydrolysate from black soldier fly larvae and schizochytrium (BSFPs) on growth performance of pet cats.

Item	CON	5% BSFPs	10% BSFPs	15% BSFPs	SEM	*p*-Value
IWB, kg	3.02	3.03	3.02	3.03	0.06	0.988
FWB, kg	3.07	3.19	3.26	3.15	0.07	0.582
ADFI, g	56.5	58.7	62.5	61.3	4.2	0.228

N = 6 (1 cat per cage). SEM, standard error of the mean; IWB, initial body weight; FWB, final body weight; ADFI, average daily feed intake; CON, group fed with basal diet; 5% BSFPs, group fed with a basal diet that replaced chicken meal, fish meal, chicken oil, and fish oil with 5% of protein hydrolysate from black soldier fly larvae and schizochytrium; 10% BSFPs, group fed with a basal diet that replaced chicken meal, fish meal, chicken oil, and fish oil with 10% of protein hydrolysate from black soldier fly larvae and schizochytrium; 15% BSFPs, group fed with a basal diet that replaced chicken meal, fish meal, chicken oil, and fish oil with 15% of protein hydrolysate from black soldier fly larvae and schizochytrium.

**Table 4:** Effects of protein hydrolysate from black soldier fly larvae and schizochytrium (BSFPs) on plasma biochemistry indicators of pet cats.

Item	Reference Interval	CON	5% BSFPs	10% BSFPs	15% BSFPs	SEM	*p*-Value
TP, g/L	60.7–95.7	68.17 ^b^	71.41^a^	70.70 ^ab^	71.54 ^a^	1.02	<0.05
ALB, g/L	28.5–39.5	20.94	21.57	20.30	21.51	0.88	0.840
GLU, mmol/L	3.63–11.89	5.85	5.85	6.52	5.42	0.57	0.259
TG, mmol/L	0.20–1.80	0.65 ^a^	0.36 ^b^	0.41 ^b^	0.36 ^b^	0.07	<0.05
TC, mmol/L	1.75–6.29	1.91	2.14	2.12	2.16	0.13	0.685
CA, mmol/L	1.65–3.23	2.07 ^b^	2.46 ^a^	2.58 ^a^	2.59 ^a^	0.11	<0.05
P, mmol/L	0.85–2.41	1.57	1.64	1.53	1.64	0.12	0.692
AST, U/L	16.0–129.0	66.50 ^a^	37.17 ^b^	21.00 ^c^	25.50 ^c^	2.04	<0.01
ALT, U/L	18.0–443.0	93.67 ^a^	38.67 ^b^	36.20 ^b^	37.50 ^b^	3.68	<0.01
CREA, umol/L	75.1–286.2	108.35 ^a^	88.02 ^b^	80.58 ^b^	78.24 ^b^	2.31	<0.05
BUN, mmol/L	5.10–11.30	11.30 ^a^	7.21 ^b^	6.55 ^b^	5.85 ^c^	0.18	<0.05

N = 6 (1 cat per cage). SEM, standard error of the mean; TP, total protein; ALB, albumin; GLU, glucose; TG, triglyceride; TC, total cholesterol; CA, calcium; P, phosphorus; AST, aspartate aminotransferase; ALT, alanine aminotransferase; CREA, creatinine; BUN, urea nitrogen. ^a–c^ Different letters on the shoulder indicate significant differences between mean values for a given behavior (*p* < 0.05).

**Table 5:** Effects of protein hydrolysate from black soldier fly larvae and schizochytrium (BSFPs) on plasma antioxidative level of pet cats.

Item	CON	5% BSFPs	10% BSFPs	15% BSFPs	SEM	*p*-Value
T-AOC, mM	1.04	1.00	1.02	1.04	0.08	0.862
GSH-PX, U/mL	1934 ^b^	2279 ^ab^	2546 ^a^	2596 ^a^	160	<0.05
SOD, U/mL	56.14 ^b^	65.61 ^a^	66.89 ^a^	65.75 ^a^	4.06	<0.05
MDA, nmol/mL	2.48 ^a^	1.69 ^b^	1.73 ^b^	1.71 ^b^	0.15	<0.05

N = 6 (1 cat per cage). SEM, standard error of the mean; T-AOC, total antioxidant capacity; GSH-PX, glutathione peroxidase; SOD, superoxide dismutase; MDA, malondialdehyde. ^a,b^ Different letters on the shoulder indicate significant differences between mean values for a given behavior (*p* < 0.05).

**Table 6:** Effects of protein hydrolysate from black soldier fly larvae and schizochytrium (BSFPs) on pro-inflammatory cytokines level of pet cats.

Item	CON	5% BSFPs	10% BSFPs	15% BSFPs	SEM	*p*-Value
TNF-α, pg/mL	12.49	12.92	11.45	14.67	1.35	0.532
IL-1β, pg/mL	7.77 ^a^	6.62 ^a^	5.22 ^b^	5.13 ^b^	0.43	<0.05
IL-8, pg/mL	18.97 ^a^	19.24 ^a^	18.85 ^a^	10.15 ^b^	1.86	<0.05

N = 6 (1 cat per cage). SEM, standard error of the mean; TNF-α, tumor necrosis factor-α; IL-1β, interleukin-1β; IL-8, interleukin-8. ^a,b^ Different letters on the shoulder indicate significant differences between mean values for a given behavior (*p* < 0.05).

**Table 7:** Effects of protein hydrolysate from black soldier fly larvae and schizochytrium (BSFPs) on plasma immune levels of pet cats.

Item	CON	5% BSFPs	10% BSFPs	15% BSFPs	SEM	*p*-Value
IgA, ug/mL	38.56 ^c^	64.01 ^a^	68.37 ^a^	46.12 ^b^	3.21	<0.05
IgG, ug/mL	1250 ^c^	1424 ^bc^	1586 ^b^	1993 ^a^	151	<0.05
IgM, ug/mL	676	763	608	529	51	0.215

N = 6 (1 cat per cage). SEM, standard error of the mean; IgA, immunoglobulin A; IgG, immunoglobulin G; IgM, immunoglobulin M. ^a–c^ Different letters on the shoulder indicate significant differences between mean values for a given behavior (*p* < 0.05).

**Table 8:** Effects of protein hydrolysate from black soldier fly larvae and schizochytrium (BSFPs) on palatability of pet cats.

Item			SEM	*p*-Value
	CON	5% BSFPs		
First sniff, %	37.50	62.50	2.98	<0.001
First bite, %	34.57	65.43	3.38	<0.001
Feed intake, g/30 min	28.35	36.51	1.93	<0.001
	CON	10% BSFPs		
First sniff, %	33.33	66.67	3.58	<0.001
First bite, %	33.33	66.67	3.58	<0.001
Feed intake, g/30 min	21.15	33.58	1.75	<0.001
	CON	15% BSFPs		
First sniff, %	34.38	65.62	3.66	<0.001
First bite, %	30.72	69.28	3.87	<0.001
Feed intake, g/30 min	23.02	36.14	2.57	<0.001

N = 6 (1 cat per cage). SEM, standard error of the mean. The extremely significant difference existed between CON group and treatments (5% BSFPs, 10% BSFPs, and 15% BSFPs) (*p* < 0.001).

## Data Availability

Data are contained within the article.
